# Function and Distribution of the Wamide Neuropeptide Superfamily in Metazoans

**DOI:** 10.3389/fendo.2020.00344

**Published:** 2020-05-28

**Authors:** Elizabeth A. Williams

**Affiliations:** Living Systems Institute, University of Exeter, Exeter, United Kingdom

**Keywords:** neuropeptide, wamide, myoinhibitory peptide, GLWamide, allatostatin B

## Abstract

The Wamide neuropeptide superfamily is of interest due to its distinctive functions in regulating life cycle transitions, metamorphic hormone signaling, and several aspects of digestive system function, from gut muscle contraction to satiety and fat storage. Due to variation among researchers in naming conventions, a global view of Wamide signaling in animals in terms of conservation or diversification of function is currently lacking. Here, I summarize the phylogenetic distribution of Wamide neuropeptides based on current data and describe recent findings in the areas of Wamide receptors and biological functions. Common trends that emerge across Cnidarians and protostomes are the presence of multiple Wamide receptors within a single organism, and the fact that Wamide signaling likely functions across an extensive variety of biological systems, including visual, circadian, and reproductive systems. Important areas of focus for future research are the further identification of Wamide-receptor pairs, confirmation of the phylogenetic distribution of Wamides through largescale sequencing and mass spectrometry, and assignment of different functions to specific subsets of Wamide-expressing neurons. More extensive study of Wamide signaling throughout larval development in a greater number of phyla is also important in order to understand the role of Wamides in hormonal regulation. Defining the evolution and function of neuropeptide signaling in animal nervous systems will benefit from an increased understanding of Wamide function and signaling mechanisms in a wider variety of organisms, beyond the traditional model systems.

## Introduction

Neuropeptides are short peptidergic molecules released by animal neurons that act as modulators or hormones to regulate biological processes. These signaling molecules are notable for being present in the nervous system of early metazoans, and for their important functions in regulating animal behavior and physiology. Historically, neuropeptides have been named according to their function, or where function is unknown, according to repetitive conserved sequence motifs found in the precursor peptide. These naming strategies have the unfortunate consequence of often obscuring neuropeptide relationships across species or phyla. The Wamide neuropeptide superfamily is a striking example of this. Wamides are repetitive proneuropeptides that contain multiple cleavage sites flanking short, amidated active peptides with a conserved C-terminal tryptophan (W). This neuropeptide superfamily is of ancient origin, already present in the last common ancestor of cnidarians and protostomes (1). Depending on the species in which they were studied, Wamides have been referred to as myoinhibitory peptide (MIP), allatostatin B (ASTB), prothoracicostatic peptide (PTSP), WWamide, GLWamide, or metamorphosin A (MMA). Even the name “Wamide” is not ideal for defining this neuropeptide family, since neuropeptides from other families may also contain a C-terminal amidated tryptophan residue. For example, adipokinetic hormone (AKH) in some insect species, molluscan APGWamides and echinoderm luqins, and some insect short neuropeptide F's (sNPF) also have C-terminal Wamide motifs, but phylogenetic analyses indicate that these neuropeptides belong to the AKH/corazonin/ACP/GnRH superfamily, the RYa/luqin, and the sNPF/prolactin superfamilies, respectively (2–5), therefore they are not considered here. In this mini review, I aim to unite recent knowledge of Wamide expression and function in diverse phyla to improve understanding of their evolutionary history and identify remaining knowledge gaps where future research could enlighten the evolution of Wamide function and mechanism of action.

## A Brief History of Wamide Discovery

The first known Wamide discovered was locust myoinhibitory peptide (LOM-MIP). LOM-MIP was identified as a suppressor of visceral muscle contraction in hindgut and oviduct in 1991 (6). Subsequently, WWamide neuropeptide was identified in the mollusc *Achatina fulica* (7) and the first cnidarian GLWamide, known as metamorphosin A (MMA), was identified in *Anthopleura elegantissima* (8). Initially, both WWamide and MMA were considered novel peptides unrelated to insect MIPs. The term allatostatin B was used to describe MIPs which inhibited juvenile hormone III synthesis in the cricket *Gryllus bimaculatus* (9). In the silkworm *Bombyx mori*, MIPs were anointed “prothoracicostatic hormone” (PTSP), due to their inhibition of ecdysone synthesis in this species (10), although cricket ASTBs were also found to inhibit ovarian ecdysteroid synthesis prior to this (11). The first crustacean Wamide was identified in crab in 2005 and was noted to be related to insect ASTBs (12).

Fifteen years after the discovery of LOM-MIP, the term “Wamides” was first used to describe this neuropeptide superfamily in the construction of a metazoan neuropeptide database by Liu et al. (13), who grouped Arthropod MIP/ASTB/PTSP, cnidarian GLWamides, molluscan WWamides and nematode MIPs within the Wamide family. Annelid Wamides were identified in 2011 by Veenstra (14), who noted their link to insect and mollusc Wamides. Genome analysis of the gastropod *Lottia gigantea* reinforced the link between molluscan WWamides and insect ASTB (15). Largescale similarity-based clustering of metazoan neuropeptides revealed that Wamides form part of the ancient central cluster of repetitive proneuropeptides that give rise to short, amidated peptides, with representative sequences from annelids, molluscs, platyhelminths, nematodes, arthropods, and cnidarians (1).

## Phylogenetic Distribution Of Wamides

**Figure 1:**
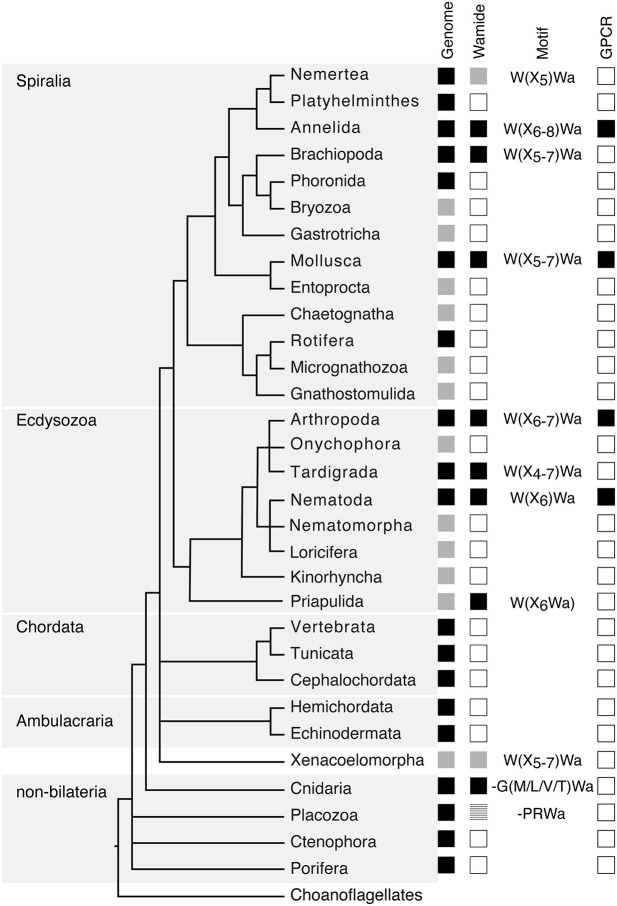
Phylogenetic distribution of Wamide neuropeptides. In “Genome” column, black boxes indicate the presence of both genome and transcriptome data, gray boxes indicate the presence of transcriptome data only, without a published genome. In “Wamide” column, black boxes indicate confirmed presence of Wamide neuropeptide, gray boxes indicate putative Wamide sequence (partial sequence, sequence evidence only from transcriptome shotgun assembly), striped gray box indicates uncertainty of Wamide superfamily orthology, white boxes indicate lack of Wamide according to currently available sequence data. “Motif” column indicates conserved structure of Wamides within each phyla. In “GPCR” column, black boxes indicate presence of MIP/sex peptide receptor GPCR ortholog confirmed by receptor deorphanization assay, white boxes indicate currently no orthologous biochemically confirmed GPCR. Phylogeny and presence of genome based on Bezares et al. (16), Figure 6, with authors' permission. Tree structure based on a phylogenomic study with Bayesian inference under the CAT + GTR + γ4 model to suppress long-branch attraction artifacts (17).

Following initial discoveries of Wamides through peptide purification and sequencing, recent largescale genomic and transcriptomic analyses have enabled a more complete view of Wamide distribution throughout the animal kingdom (Figure 1). Wamide neuropeptides also occur in brachiopods (18), tardigrades and priapulids (19–21). Transcriptome analysis of xenacoelomorph neuropeptides did not find myoinhibitory peptide orthologs, although a putative MIP receptor was found in an acoel (22). A transcript fragment of a MIP can be found however in a *Xenoturbella bockii* transcriptome dataset used to identify homeobox genes (21), thus the presence of MIP signaling in xenacoelomorphs remains uncertain for now. Extensive transcriptome analyses recently confirmed the presence of Wamides in all molluscan groups, but also supported the absence of Wamides (ASTB) in phoronids, platyhelminths and rotifers, as well as ectoprocts and entoprocts (23).

Despite extensive sequencing data, Wamides have not been identified in deuterostomes, although phylogenetic analysis of GPCRs identified orphan human GPCR139 and GPCR142 as potential MIP receptor orthologs (24). Ligands for these receptors have not been confirmed *in vivo* but are suggested to be small peptides or amino acids, such as L-Trp (W) and L-Phe (F) (25, 26). Therefore, while a Wamide signaling system arose early in metazoan evolution, in a cnidarian/bilaterian ancestor, it appears to have been lost multiple times during evolution, including in ambulacrarians and tunicates. This trend also occurs within phyla, for example, although widespread among insects, Wamides have not been found in the honey bee, wasp, or leaf-cutting ants (27, 28). A precise picture of Wamide gain and loss throughout metazoan evolution requires further sequence data, particularly from lesser-studied spiralian and ecdysozoan phyla.

**Figure 2:**
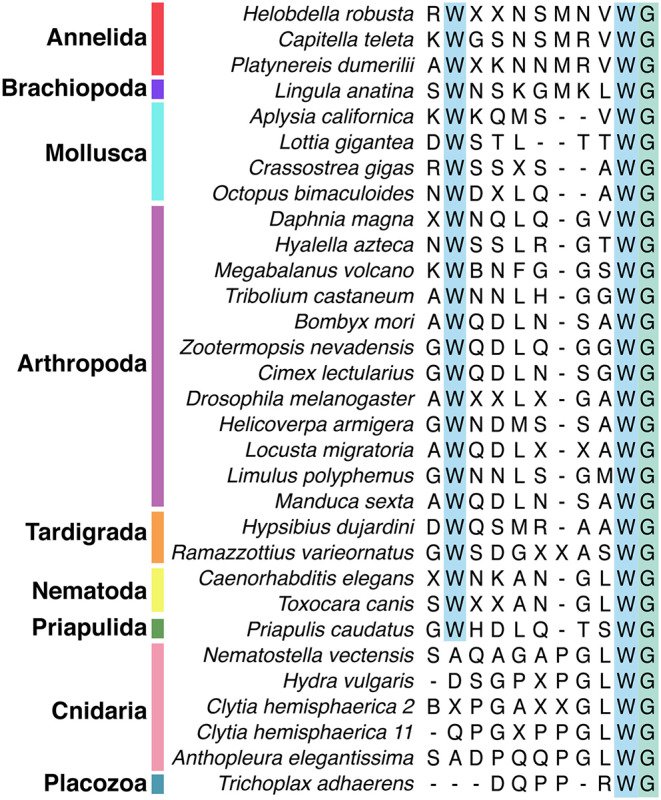
Alignment of subset of mature Wamide consensus sequences from representative species of each phyla. Conserved N- and C-terminal tryptophan residues are highlighted (blue). The terminal glycine (green) is modified to provide an amide group during posttranslational modification. Sequences used to generate consensus are contained in Supplementary Data and available through NCBI.

Wamide distribution among early branching metazoan phyla is of ongoing interest. Recent bioinformatic analyses indicate that among cnidarians, GLWamides are absent in class Scyphozoa, Staurozoa, and Octocorallia (29). The same analysis uncovers new G{A/V/T}Wamides in Cubozoa, Scyphozoa, and Staurozoa. How these new cnidarian Wamides relate to GLWamides and the rest of the Wamide neuropeptide superfamily awaits further investigation, however it is interesting to note that an {A/V/T}Wamide C-terminal motif also occurs in arthropod, mollusc and annelid Wamides (Figure 2). In the placozoan *Trichoplax adhaerens*, an RWamide precursor was found through bioinformatic prediction (30). Placozoan RWamide has strong resemblance to cnidarian GLWamides (Figure 2), however since some luqin/RYamide and sNPF/prolactin family neuropeptides also show a conserved RWamide C-terminal motif (4, 5), it is currently unclear to which superfamily the placozoan RWamide belongs. Similarly, although putative neuropeptide precursors were found in ctenophores these did not have homology to other metazoan neuropeptides. However, since the cross-phylum conservation of neuropeptides can be limited to just a few residues (1, 31), the existence of Ctenophore Wamides remains a possibility. No neuropeptide-like precursors have been identified in poriferans to date, despite neuropeptide-processing enzymes being present in the *Amphimedon queenslandica* genome (32). Further peptide and receptor characterization through mass spectrometry, largescale receptor deorphanization and functional studies are needed to clarify the occurrence and evolution of Wamide signaling in poriferans, ctenophores, placozoans, and cnidarians.

## Wamide Receptors

The most well-described Wamide receptor is a rhodopsin family G-protein-coupled receptor (GPCR). This was initially called the sex peptide receptor (SPR) after its first characterized ligand, the *Drosophila* sex peptide (33). Whereas sex peptides only occur in the family Drosophilidae and specifically activate the *Drosophila* receptor, *Drosophila* MIPs can activate orthologous receptors from other insect groups and from *Aplysia californica*, a mollusc (34). Following this discovery, it was deduced that the sex peptide receptor's ancestral ligands were in fact MIPs (34, 35), causing a reassignment of name to myoinhibitory peptide receptor. Sex peptides differ significantly from MIPs in their primary amino acid sequence; they are larger (36aa cf. 9-12aa), lack C-terminal amidation, and contain a disulfide bridge. Like MIPs, however, sex peptides contain a pair of tryptophan (W) residues which are predicted to stabilize a beta-turn secondary structure adopted by both peptides (34). The conserved tryptophans are required for receptor binding in both sex peptide and MIPs, indicating that they may interact with a common binding site on the receptor (34).

Orthologs of the MIP receptor have been biochemically characterized in insects [fruitfly, mosquito (34), tick (36), kissing bug (37), silkworm (35)], a mollusc (34), nematode (38), and annelids (39). Receptor deorphanization assays with modified peptides show that the two conserved tryptophan residues are important for receptor activation. The C-terminal tryptophan residue is especially critical for receptor activation; when this residue is replaced with an alanine, all receptor activity is lost in both annelids and insects (34, 37, 39, 40).

Unlike protostomes, a cnidarian Wamide receptor has not yet been identified. Receptor identification based on phylogenetic analyses alone may prove difficult, as most cnidarian GPCRs are more closely related to each other than to specific nephrozoan receptors (22). A largescale combinatorial receptor de-orphanization approach, such as that used to identify several novel peptide-receptor pairs in *Platynereis dumerilii* (41), would be useful in this endeavor. Without a known receptor, cnidarian Wamides can not be definitively confirmed as orthologs of protostome Wamides. However, sequence similarity clustering (1), the conservation of the C-terminal GLWamide motif in nematode and some insect MIPs (Figure 2 and Supplementary Data), the greater importance of the C-terminal tryptophan for receptor binding (see above), and the occasional occurrence of an N-terminal tryptophan in cnidarian GLWamides (e.g., *Hydra vulgaris*, Supplementary Data) are in support of orthology.

The presence of multiple Wamide receptors in the same organism is emerging as a common theme. *Caenorhabditis elegans* has three receptors related to arthropod MIP receptors (38). In *Drosophila melanogaster*, loss of the MIP/SPR GPCR does not affect the function of MIP in appetite control or female mating behavior, indicating that MIP may act through one or more additional receptors (42, 43). In the hydrozoan *Hydra magnipapillata*, the GLWamide Hym-248 activates both endodermal muscle contraction and sphincter muscle contraction, whereas other GLWamides activate only sphincter muscle contraction (44). This additional function of Hym-248 suggests that a receptor specific only to this peptide is expressed in endodermal muscle, while a more generalist GLWamide receptor is expressed in sphincter muscle (45). These findings indicate that a common evolutionary strategy for the diversification of Wamide peptide function is through the addition of receptors with varying binding specificities.

A new family of Wamide receptors has recently been identified in the marine worm *Platynereis dumerilii* (46). These receptors are peptide-gated ion channels from the degenerin/epithelial sodium channel family. The MIP-gated ion channel (MGIC) receptor is paralagous to the FMRFamide-gated sodium channels found in snails and cnidarians (47, 48). The mechanism of MIPs binding to the MGIC receptor is similar to that of binding to the MIP GPCR in that the conserved tryptophans are essential for receptor activation. As with the MIP GPCR, replacement of the N-terminal tryptophan with an alanine reduced MGIC receptor activity, while replacement of the C-terminal tryptophan abolished MGIC receptor activity (46). Although all eleven mature MIP peptides from the same precursor activate both the MIP GPCR and the MGIC receptor, each mature peptide preferentially activates one of the receptor types, suggesting a mechanism for diversification of peptide function. Comparison of *in vivo* concentrations of the different mature MIPs to receptor activation concentrations determined *in vitro* would indicate whether different mature MIPs really activate both MGIC and GPCR receptor types *in vivo*. Phylogenetic analysis of peptide-gated ion channels identified nematode, amphioxus, and cnidarian sequences related to MGIC/FaNaC peptide-gated ion channels (46), however these putative peptide-gated ion channels are yet to be assigned a peptidergic ligand through physiological assays.

## Known Functions of Wamides

Throughout metazoans, Wamides have a wide variety of functions and often carry out multiple functions within the same organism. One known function of Wamides shared between cnidarians and protostomes is the regulation of life cycle transitions. Cnidarian GLWamides induce larval settlement and metamorphosis in the hydroid *Hydractinia echinata*, as well as the larvae of several coral species, and the hydrozoan *Clytia hemisphaerica* (8, 49–52). Knockdown of the GLWamide precursor gene in *Nematostella vectensis* showed that GLWamide is not necessary for metamorphosis, at least in this species, but plays a modulatory role in determining metamorphic timing (53). Similar to cnidarians, treatment of larvae of *Platynereis dumerilii* with synthetic MIP peptide induces settlement (39). Both cnidarian and *P. dumerilii* Wamide-expressing cells are sensory-neurosecretory, suggesting that this neuropeptide plays a role in activating a settlement and metamorphosis program in response to specific environmental cues, however the nature of the environmental cues that trigger Wamide peptide release, and the downstream signaling pathways activated or repressed by Wamides require further characterization. Microarray and RNA-Seq studies of GLWamide-treated branching coral *Acropora millepora* identified significant changes in transcription after peptide exposure (54, 55). These studies may be useful for identifying candidate genes and pathways for functional investigations of Wamide signaling networks acting in coral metamorphosis, now that tools for genome editing are available for coral (56). Curiously, the *Hydra* GLWamide, Hym-248, also promotes settlement and metamorphosis in two species of sponge (57). This suggests that although Wamides haven't been identified in sponges, there may be some overlap between the signal transduction pathways involved in sponge and cnidarian metamorphosis, particularly at the level of neuropeptide receptors.

Similar to regulating marine invertebrate larval settlement and metamorphosis, in some insect species, Wamides regulate levels of metamorphic hormones. In the silkworm, PTSPs suppress ecdysteroidogenesis in the prothoracic glands (10, 35, 58), while in crickets, allatostatin B inhibits juvenile hormone production (59, 60). MIPs also regulate aspects of the behavioral sequence underlying ecdysis in *D. melanogaster* and *Manduca sexta*, as part of a peptidergic signaling cascade initiated by ecdysis triggering hormone (61–64). However, functions of Wamides during insect larval development and how Wamides regulate hormone production or release still require further investigation. Do Wamides also regulate hormones similar to juvenile hormone or ecdysone in the induction of marine invertebrate metamorphosis? Juvenile hormone and its precursor, methyl farnesoate, can regulate larval metamorphosis in polychaetes and barnacles (65, 66) and ecdysone signaling may play a role in crustacean and mollusc metamorphosis (67), however Wamide function in mollusc and crustacean larval stages has not yet been reported, nor has the effect of Wamides on specific marine invertebrate hormones.

Perhaps the most widely conserved function of Wamides is the regulation of muscle contraction. In arthropods, MIPs inhibit muscle contraction in the hindgut, oviduct and heart/pyloric system (6, 68–72). Contrary to their name, MIPs promote muscle contractions in annelid gut muscles (73). Cnidarian GLWamides also promote muscle contractions in the longitudinal (ectodermal) muscles and circumferential (endoderm) muscles (44). A specialization of the muscle contraction function of some *Hydra* GLWamides is in inducing the contraction of sphincter muscles, which causes detachment of buds from a parental polyp, thereby linking Wamides to the regulation of asexual reproduction in *Hydra* (74). Mollusc WWamides inhibit the phasic contractions of the anterior byssus retractor muscle in mussel, but potentiate contractions of the penis and radula retractor muscles in land snail, as well as inducing contractions of the radula protractor muscle in a gastropod (7). Studies of the effects of Wamides on muscle contraction have revealed that Wamide effects are dependent on the current physiological state of the system (71), the concentration of peptide applied (7, 75), the type of receptor activated (45), and whether the peptide acts pre- or post-synaptically (7).

Additional to the regulation of muscle contraction in the digestive system, Wamides have been implicated in a variety of feeding-related functions, primarily in insects. In the kissing bug and ticks, MIP expression in the salivary glands suggests a role in salivation (36, 37, 76, 77). Activating MIP neurons in *D. melanogaster* adults decreased food intake and body weight and reduced the sensitivity of starved flies toward food (42). Also in *D. melanogaster*, MIPs modulate attraction to polyamine food odors in mated females (78). This sex-specific modulation is through an autocrine signaling mechanism, with MIP and the MIP/sex peptide receptor both expressed in taste and olfactory neurons. Recent findings from *C. elegans* show a role for MIP in aversive gustatory short-term learning, and long-term learning of salt avoidance behaviors (38). MIP's function in sensing specific food cues in adult fly and nematode shows interesting parallels with the role of MIP in regulating marine invertebrate metamorphosis. In both cases, MIP is expressed in neurons with chemosensory morphology and detection of specific cues and release of MIPs activates a switch in states. Cues for marine invertebrate metamorphosis are also often associated with preferred food sources of their future adult stages (79).

Beyond the regulation of life cycle transitions, muscle contraction and feeding/digestive, MIPs may also play a role in diverse biological systems, including reproductive, visual, and circadian systems. In female *D. melanogaster*, MIP-expressing abdominal interneurons enhance mating receptivity in mated females (43). Additionally, MIP-expressing interneurons in the central brain form part of a mechanosensory circuit that informs female mating decisions (80). Also in *D. melanogaster*, some optic lobe neurons express MIP, although it is not yet clear if these regulate signaling in the visual or circadian system, or both (81). MIP expression in *D. melanogaster* cycles with circadian rhythm, in line with the role of MIP in maintaining a sleep-like state in adults (82). Spatial expression of MIP in cockroach brains also suggests a role in the circadian system (83, 84). MIP expression in insects has some similarities to GLWamide expression in jellyfish. For example, GLWamide expression is seen in the gonadal ectoderm cells of *Clytia hemisphaerica* and *Cytaeis uchidae* (85, 86), as well as the photoreceptive organs of the jellyfish *Cladonema radiatum, Aurelia aurita*, and *Tripedalia cystophora* (87). In both insects and cnidarians, further functional studies are needed to reveal the function of Wamides in reproduction, vision or circadian rhythm. These previous studies of spatial expression highlight the usefulness of detailed expression atlases that encompass different life cycle stages, sexes and whole-body analyses for additional species and phyla, to provide initial indications of Wamide function in distinct biological systems.

## Conclusions and Future Directions

Wamides are clearly neuropeptides of significant importance to nervous system signaling, with a role in diverse biological systems throughout an organism's life cycle. The fact that Wamide signaling is lost in some species or phyla indicates that Wamides function within more complex networks of neuropeptide signaling, sometimes playing a modulatory but non-essential role, and their function may be taken on or replaced by other neuropeptides. Several similarities are seen in both the function and spatial expression of Wamides in cnidarians and protostomes, supporting the definition of this neuropeptide superfamily, however the identification of cnidarian Wamide receptors will further enlighten the evolution of Wamide signaling in metazoans.

The most detailed studies of Wamide signaling to date have been carried out in model organisms *D. melanogaster* and *C. elegans* and these studies show that subsets of MIP-expressing neurons are likely different cell types (e.g., sensory neurons vs. interneurons) responsible for different aspects of Wamide function. It is therefore important for future studies aiming to uncover mechanisms of Wamide signaling to develop methods for manipulating specific subsets of Wamide-expressing neurons. One approach to this is through the development of libraries of reporter constructs driven by different promoters with a range of cell-specificities. Further understanding of Wamide signaling can also be achieved by analysis of genes co-expressed in Wamide- and Wamide receptor-expressing cells and more widespread analyses of Wamide receptor expression. These analyses can indicate mechanisms of signaling, such as autoregulation in cells expressing both Wamide and receptor, or association with specific neurotransmitters, such as GABA or acetylcholine. They can also be used to generate maps of potential signaling cascades, as in insect ecdysis (61), through the comparison of expression of other neuropeptides and receptors. Receptor-ligand expression mapping based on single cell transcriptome data, as in (88), which should then be functionally tested, will facilitate these analyses.

Another aspect of Wamide signaling of importance for future studies is the identification of signaling cascades activated following Wamide release and receptor binding, in terms of which class of G protein is recruited, if indeed the Wamide signal is GPCR-mediated, and which downstream signaling pathways are activated or repressed. Again, single cell RNA-Seq analyses or precise spatial expression mapping can be used to identify which elements of the signaling pathway are present in the target cells of Wamide signaling. With both distinctive and conserved functions, the Wamide superfamily is an excellent model for studying the evolution of neuropeptide signaling and patterns of peptide-receptor coevolution in animal nervous systems.

## Author Contributions

EW conceived the study and wrote the paper.

## Conflict of Interest

The author declares that the research was conducted in the absence of any commercial or financial relationships that could be construed as a potential conflict of interest.

## References

[1] Jekely G. Global view of the evolution and diversity of metazoan neuropeptide signaling. Proc Natl Acad Sciences USA. (2013) 110:8702–7. 10.1073/pnas.1221833110 23637342 PMC3666674

[2] Tian S Zandawala M Beets I Baytemur E Slade SE Scrivens JH. Urbilaterian origin of paralogous GnRH and corazonin neuropeptide signalling pathways. Sci Rep. (2016) 6:28788. 10.1038/srep28788 27350121 PMC4923880

[3] Zandawala M Tian S Elphick MR. The evolution and nomenclature of GnRH-type and corazonin-type neuropeptide signaling systems. Gen Comp Endocrinol. (2018) 264:64–77. 10.1016/j.ygcen.2017.06.007 28622978

[4] Yañez-Guerra LA Delroisse J Barreiro-Iglesias A Slade SE Scrivens JH Elphick MR. Discovery and functional characterisation of a luqin-type neuropeptide signalling system in a deuterostome. Sci Rep. (2018) 8:7220. 10.1038/s41598-018-25606-2 29740074 PMC5940834

[5] Yañez-Guerra LA Zhong X Moghul I Butts T Zampronio CG Jones AM Urbilaterian origin and evolution of sNPF-type neuropeptide signaling. BioRxiv. (2019). 10.1101/712687

[6] Schoofs L Holman GM Hayes TK Nachman RJ De Loof A. Isolation, identification and synthesis of locusta myoinhibiting peptide (LOM-MIP), a novel biologically active neuropeptide from locusta migratoria. Regul Pept. (1991) 36:111–9. 10.1016/0167-0115(91)90199-Q 1796179

[7] Minakata H Ikeda T Muneoka Y Kobayashi M Nomoto K. WWamide-1,−2 and−3: novel neuromodulatory peptides isolated from ganglia of the African giant snail, achatina fulica. FEBS Lett. (1993) 323:104–8. 10.1016/0014-5793(93)81458-C 8495720

[8] Leitz T Morand K Mann M. Metamorphosin A: a novel peptide controlling development of the lower metazoan hydractinia echinata (coelenterata, hydrozoa). Dev Biol. (1994) 163:440–6. 10.1006/dbio.1994.1160 7911112

[9] Lorenz MW Kellner R Hoffmann KH. A family of neuropeptides that inhibit juvenile hormone biosynthesis in the cricket, gryllus bimaculatus. J Biol Chem. (1995) 270:21103–8. 10.1074/jbc.270.36.21103 7673141

[10] Hua YJ Tanaka Y Nakamura K Sakakibara M Nagata S Kataoka H. Identification of a prothoracicostatic peptide in the larval brain of the silkworm, bombyx mori. J Biol Chem. (1999) 274:31169–73. 10.1074/jbc.274.44.31169 10531308

[11] Lorenz MW Lorenz JI Treiblmayr K Hoffmann K-H *In vivo* effects of allatostatins in crickets, gryllus bimaculatus (ensifera: gryllidae). Arch Insect Biochem Physiol. (1998) 38:32–43. 10.1002/(SICI)1520-632738:1<32::AID-ARCH4>3.0.CO;2-X

[12] Fu Q Li L. *De novo* sequencing of neuropeptides using reductive isotopic methylation and investigation of ESI QTOF MS/MS fragmentation pattern of neuropeptides with N-terminal dimethylation. Anal Chem. (2005) 77:7783–95. 10.1021/ac051324e 16316189

[13] Liu F Baggerman G Schoofs L Wets G. The construction of a bioactive peptide database in metazoa. J Proteome Res. (2008) 7:4119–31. 10.1021/pr800037n 18707150

[14] Veenstra JA. Neuropeptide evolution: neurohormones and neuropeptides predicted from the genomes of capitella teleta and helobdella robusta. Gen Comp Endocrinol. (2011) 171:160–75. 10.1016/j.ygcen.2011.01.005 21241702

[15] Veenstra JA. Neurohormones and neuropeptides encoded by the genome of lottia gigantea, with reference to other mollusks and insects. Gen Comp Endocrinol. (2010) 167:86–103. 10.1016/j.ygcen.2010.02.010 20171220

[16] Bezares-Calderón LA Berger J Jékely G. Diversity of cilia-based mechanosensory systems and their functions in marine animal behaviour. Phil Trans R Soc B Biol Sci. (2020) 375:20190376. 10.1098/rstb.2019.0376 31884914 PMC7017336

[17] Laumer CE Fernández R Lemer S Comsbosch D Kocot KM Riesgo A Revisiting metazoan phylogeny with genomic sampling of all phyla. Phil Trans R Soc B Biol Sci. (2019) 286:20190831 10.1098/rspb.2019.0831 PMC6650721 31288696

[18] Luo Y-J Takeuchi T Koyanagi R Yamada L Kanda M Khalturina M. The lingula genome provides insights into brachiopod evolution and the origin of phosphate biomineralization. Nat Commun. (2015) 6:8301. 10.1038/ncomms9301 26383154 PMC4595640

[19] Koziol U. Precursors of neuropeptides and peptide hormones in the genomes of tardigrades. Gen Comp Endocrinol. (2018) 267:116–27. 10.1016/j.ygcen.2018.06.012 29935140

[20] Borner J Rehm P Schill RO Ebersberger I Burmester T. A transcriptome approach to ecdysozoan phylogeny. Mol Phylogenet Evol. (2014) 80:79–87. 10.1016/j.ympev.2014.08.001 25124096

[21] Brauchle M Bilican A Eyer C Bailly X Martínez P Ladurner P. Xenacoelomorpha survey reveals that all 11 animal homeobox gene classes were present in the first bilaterians. Genome Biol Evol. (2018) 10:2205–17. 10.1093/gbe/evy170 30102357 PMC6125248

[22] Thiel D Franz-Wachtel M Aguilera F Hejnol A Xenacoelomorph neuropeptidomes reveal a major expansion of neuropeptide systems during early bilaterian evolution. Mol Biol Evol. (2018) 35:2528–43. 10.1093/molbev/msy160

[23] De Oliveira AL Calcino A Wanninger A. Extensive conservation of the proneuropeptide and peptide prohormone complement in mollusks. Sci Rep. (2019) 9:4846. 10.1038/s41598-019-40949-0 30890731 PMC6425005

[24] Mirabeau O Joly J-S. Molecular evolution of peptidergic signaling systems in bilaterians. Proc Natl Acad Sci USA. (2013) 110:E2028–37. 10.1073/pnas.1219956110 23671109 PMC3670399

[25] Süsens U Hermans-Borgmeyer I Urny J Schaller HC. Characterisation and differential expression of two very closely related G-protein-coupled receptors, GPR139 and GPR142, in mouse tissue and during mouse development. Neuropharmacology. (2006) 50:512–20. 10.1016/j.neuropharm.2005.11.003 16378626

[26] Isberg V Andersen KB Bisig C Dietz GPH Bräuner-Osborne H Gloriam DE. Computer-Aided discovery of aromatic l-α-amino acids as agonists of the orphan g protein-coupled receptor GPR139. J Chem Inform Model. (2014) 54:1553–7. 10.1021/ci500197a 24826842

[27] Hauser F Neupert S Williamson M Predel R Tanaka Y Grimmelikhuijzen CJP. Genomics and peptidomics of neuropeptides and protein hormones present in the parasitic wasp nasonia vitripennis. J Proteome Res. (2010) 9:5296–310. 10.1021/pr100570j 20695486

[28] Nygaard S Zhang G Schiøtt M Li C Wurm Y Hu H. The genome of the leaf-cutting ant acromyrmex echinatior suggests key adaptations to advanced social life and fungus farming. Genome Res. (2011) 21:1339–48. 10.1101/gr.121392.111 21719571 PMC3149500

[29] Koch TL Grimmelikhuijzen CJP. Global neuropeptide annotations from the genomes and transcriptomes of cubozoa, scyphozoa, staurozoa (cnidaria: medusozoa), and octocorallia (cnidaria: anthozoa). Front Endocrinol. (2019) 10:831. 10.3389/fendo.2019.00831 31866941 PMC6909153

[30] Nikitin M. Bioinformatic prediction of Trichoplax adhaerens regulatory peptides. Gen Comp Endocrinol. (2015) 212:145–55. 10.1016/j.ygcen.2014.03.049 24747483

[31] Jékely G Paps J Nielsen C. The phylogenetic position of ctenophores and the origin(s) of nervous systems. Evodevo. (2015) 6:1. 10.1186/2041-9139-6-1 25905000 PMC4406211

[32] Srivastava M Simakov O Chapman J Fahey B Gauthier MEA Mitros T. The amphimedon queenslandica genome and the evolution of animal complexity. Nature. (2010) 466:720–6. 10.1038/nature09201 20686567 PMC3130542

[33] Yapici N Kim Y-J Ribeiro C Dickson BJ. A receptor that mediates the post-mating switch in drosophila reproductive behaviour. Nature. (2008) 451:33–7. 10.1038/nature06483 18066048

[34] Kim Y-J Bartalska K Audsley N Yamanaka N Yapici N Lee J-Y. MIPs are ancestral ligands for the sex peptide receptor. Proc Natl Acad Sci USA. (2010) 107:6520–5. 10.1073/pnas.0914764107 20308537 PMC2851983

[35] Yamanaka N Hua Y-J Roller L Spalovská-Valachová I Mizoguchi A Kataoka H. Bombyx prothoracicostatic peptides activate the sex peptide receptor to regulate ecdysteroid biosynthesis. Proc Natl Acad Sci USA. (2010) 107:2060–5. 10.1073/pnas.0907471107 20133850 PMC2836647

[36] Simo L Koči J Park Y. Receptors for the neuropeptides, myoinhibitory peptide and SIFamide, in control of the salivary glands of the blacklegged tick Ixodes scapularis. Insect Biochem Mol Biol. (2013) 43:376–87. 10.1016/j.ibmb.2013.01.002 23357681 PMC3602366

[37] Paluzzi J-PV Haddad AS Sedra L Orchard I Lange AB. Functional characterization and expression analysis of the myoinhibiting peptide receptor in the chagas disease vector, rhodnius prolixus. Mol Cell Endocrinol. (2015) 399:143–53. 10.1016/j.mce.2014.09.004 25218475

[38] Peymen K Watteyne J Borghgraef C Van Sinay E Beets I Schoofs L. Myoinhibitory peptide signaling modulates aversive gustatory learning in Caenorhabditis elegans. PLoS Genet. (2019) 15:e1007945. 10.1371/journal.pgen.1007945 30779740 PMC6380545

[39] Conzelmann M Williams EA Tunaru S Randel N Shahidi R Asadulina A. Conserved MIP receptor-ligand pair regulates platynereis larval settlement. Proc Natl Acad Sci USA. (2013) 110:8224–9. 10.1073/pnas.1220285110 23569279 PMC3657792

[40] Vandersmissen HP Nachman RJ Vanden Broeck J. Sex peptides and MIPs can activate the same G protein-coupled receptor. Gen Comparative Endocrinol. (2013) 188:137–43. 10.1016/j.ygcen.2013.02.014 23453963

[41] Bauknecht P Jékely G. Large-scale combinatorial deorphanization of platynereis neuropeptide GPCRs. Cell Rep. (2015) 12:684–93. 10.1016/j.celrep.2015.06.052 26190115

[42] Min S Chae H-S Jang Y-H Choi S Lee S Jeong YT. Identification of a peptidergic pathway critical to satiety responses in drosophila. Curr Biol. (2016) 26:814–20. 10.1016/j.cub.2016.01.029 26948873

[43] Jang Y-H Chae H-S Kim Y-J. Female-specific myoinhibitory peptide neurons regulate mating receptivity in drosophila melanogaster. Nat Commun. (2017) 8:1630. 10.1038/s41467-017-01794-9 29158481 PMC5696375

[44] Takahashi T Kobayakawa Y Muneoka Y Fujisawa Y Mohri S Hatta M. Identification of a new member of the GLWamide peptide family: physiological activity and cellular localization in cnidarian polyps. Comp Biochem Physiol B Biochem Mol Biol. (2003) 135:309–24. 10.1016/S1096-4959(03)00088-5 12798941

[45] Takahashi T Hatta M. The importance of glwamide neuropeptides in cnidarian development and physiology. J Amino Acids. (2011) 2011:1–8. 10.4061/2011/424501 22312460 PMC3268022

[46] Schmidt A Bauknecht P Williams EA Augustinowski K Gründer S Jékely G. Dual signaling of wamide myoinhibitory peptides through a peptide-gated channel and a GPCR in Platynereis. FASEB J. (2018) 32:5338–49. 10.1096/fj.201800274R 29688813

[47] Lingueglia E Champigny G Lazdunski M Barbry P. Cloning of the amiloride-sensitive FMRFamide peptide-gated sodium channel. Nature. (1995) 378:730–3. 10.1038/378730a0 7501021

[48] Golubovic A Kuhn A Williamson M Kalbacher H Holstein TW Grimmelikhuijzen CJP. A peptide-gated ion channel from the freshwater polyp hydra. J Biol Chem. (2007) 282:35098–103. 10.1074/jbc.M706849200 17911098

[49] Iwao K Fujisawa T Hatta M A cnidarian neuropeptide of the GLWamide family induces metamorphosis of reef-building corals in the genus acropora. Coral Reefs. (2002) 21:127–9. 10.1007/s00338-002-0219-8

[50] Erwin PM Szmant AM Settlement induction of Acropora palmata planulae by a GLW-amide neuropeptide. Coral Reefs. (2010) 29:929–39. 10.1007/s00338-010-0634-1

[51] Momose T De Cian A Shiba K Inaba K Giovannangeli C Concordet J-P. High doses of CRISPR/Cas9 ribonucleoprotein efficiently induce gene knockout with low mosaicism in the hydrozoan clytia hemisphaerica through microhomology-mediated deletion. Sci Rep. (2018) 8:11734. 10.1038/s41598-018-30188-0 30082705 PMC6078951

[52] Lechable M Jan A Weissbourd B Uveira J Gissat L Collet S An improved whole life cycle culture protocol for the hydrozoan genetic model Clytia. Hemisphaerica. (2019). 10.1101/852632 PMC7657476 32994186

[53] Nakanishi N Martindale MQ. CRISPR knockouts reveal an endogenous role for ancient neuropeptides in regulating developmental timing in a sea anemone. Elife. (2018) 7:e39742. 10.7554/eLife.39742 30223943 PMC6152798

[54] Grasso LC Negri AP Fôret S Saint R Hayward DC Miller DJ. The biology of coral metamorphosis: molecular responses of larvae to inducers of settlement and metamorphosis. Dev Biol. (2011) 353:411–9. 10.1016/j.ydbio.2011.02.010 21338599

[55] Meyer E Aglyamova GV Matz MV. Profiling gene expression responses of coral larvae (Acropora millepora) to elevated temperature and settlement inducers using a novel RNA-Seq procedure. Mol Ecol. (2011) 20:3599–616. 10.1111/j.1365-294X.2011.05205.x 21801258

[56] Cleves PA Strader ME Bay LK Pringle JR Matz MV. CRISPR/Cas9-mediated genome editing in a reef-building coral. Proc Natl Acad Sci USA. (2018) 115:5235–40. 10.1073/pnas.1722151115 29695630 PMC5960312

[57] Whalan S Webster NS Negri AP. Crustose coralline algae and a cnidarian neuropeptide trigger larval settlement in two coral reef sponges. PLoS ONE. (2012) 7:e30386. 10.1371/journal.pone.0030386 22295083 PMC3266265

[58] Liu X Tanaka Y Song Q Xu B Hua Y. Bombyx mori prothoracicostatic peptide inhibits ecdysteroidogenesis *in vivo*. Arch Insect Biochem Physiol. (2004) 56:155–61. 10.1002/arch.20005 15274176

[59] Bendena WG Donly BC Tobe SS. Allatostatins: a growing family of neuropeptides with structural and functional diversity. Ann NY Acad Sci. (1999) 897:311–29. 10.1111/j.1749-6632.1999.tb07902.x 10676459

[60] Stay B Tobe SS Bendena WG Allatostatins: identification, primary structures, functions and distribution. Adv. Insect Physiol. (1995) 267–337. 10.1016/S0065-2806(08)60066-1

[61] Kim Y-J Zitnan D Galizia CG Cho K-H Adams ME. A command chemical triggers an innate behavior by sequential activation of multiple peptidergic ensembles. Curr Biol. (2006) 16:1395–407. 10.1016/j.cub.2006.06.027 16860738

[62] Kim Y-J Kim Y Zitnan D Cho K Schooley DA Mizoguchi A. Central peptidergic ensembles associated with organization of an innate behavior. Proc Natl Acad Sci USA. (2006) 103:14211–6. 10.1073/pnas.0603459103 16968777 PMC1599936

[63] Santos JG Vömel M Struck R Homberg U Nässel DR Wegener C. Neuroarchitecture of peptidergic systems in the larval ventral ganglion of drosophila melanogaster. PLoS ONE. (2007) 2:e695. 10.1371/journal.pone.0000695 17668072 PMC1933254

[64] Davis NT. Localization of myoinhibitory peptide immunoreactivity in manduca sexta and bombyx mori, with indications that the peptide has a role in molting and ecdysis. J Exp Biol. (2003) 206:1449–60. 10.1242/jeb.00234 12654884

[65] Laufer H Biggers WJ Unifying concepts learned from methyl farnesoate for invertebrate reproduction and post-embryonic development. Am Zool. (2001) 41:442–57. 10.1093/icb/41.3.442

[66] Yamamoto H Okino T Yoshimura E Tachibana A Shimizu K Fusetani N Methyl farnesoate induces larval metamorphosis of the barnacle, balanus amphitrite via protein kinase C activation. J Exp Zool. (1997) 278:349–55. 10.1002/(SICI)1097-010X(19970815)278:6<349::AID-JEZ2>3.0.CO;2-O

[67] Joyce A Vogeler S Molluscan bivalve settlement and metamorphosis: neuroendocrine inducers and morphogenetic responses. Aquaculture. (2018) 487:64–82. 10.1016/j.aquaculture.2018.01.002

[68] Blackburn MB Wagner RM Kochansky JP Harrison DJ Thomas-Laemont P Raina AK. The identification of two myoinhibitory peptides, with sequence similarities to the galanins, isolated from the ventral nerve cord of manduca sexta. Regul Pept. (1995) 57:213–9. 10.1016/0167-0115(95)00034-9 7480870

[69] Predel R Rapus J Eckert M. Myoinhibitory neuropeptides in the american cockroach. Peptides. (2001) 22:199–208. 10.1016/S0196-9781(00)00383-1 11179813

[70] Aguilar R Maestro JL Belles X Effects of myoinhibitory peptides on food intake in the German cockroach. Physiol Entomol. (2006) 31:257–61. 10.1111/j.1365-3032.2006.00513.x

[71] Szabo TM Chen R Goeritz ML Maloney RT Tang LS Li L. Distribution and physiological effects of B-type allatostatins (myoinhibitory peptides, MIPs) in the stomatogastric nervous system of the crab Cancer borealis. J Comp Neurol. (2011) 519:2658–76. 10.1002/cne.22654 21491432 PMC3245975

[72] Fu Q Tang LS Marder E Li L. Mass spectrometric characterization and physiological actions of VPNDWAHFRGSWamide, a novel B type allatostatin in the crab, cancer borealis. J Neurochem. (2007) 101:1099–107. 10.1111/j.1471-4159.2007.04482.x 17394556

[73] Williams EA Conzelmann M Jékely G. Myoinhibitory peptide regulates feeding in the marine annelid Platynereis. Front Zool. (2015) 12:1. 10.1186/s12983-014-0093-6 25628752 PMC4307165

[74] Takahashi T Muneoka Y Lohmann J Lopez de Haro MS Solleder G Bosch TC. Systematic isolation of peptide signal molecules regulating development in hydra: LWamide and PW families. Proc Natl Acad Sci USA. (1997) 94:1241–6. 10.1073/pnas.94.4.1241 9037037 PMC19775

[75] Katsukura Y Ando H David CN Grimmelikhuijzen CJP Sugiyama T. Control of planula migration by LWamide and RFamide neuropeptides in *Hydractinia echinata*. J Exp Biol. (2004) 207:1803–10. 10.1242/jeb.00974 15107436

[76] Simo L Zitnan D Park Y. Two novel neuropeptides in innervation of the salivary glands of the black-legged tick, ixodes scapularis: myoinhibitory peptide and SIFamide. J Comp Neurol. (2009) 517:551–63. 10.1002/cne.22182 19824085 PMC3577097

[77] Lange AB Alim U Vandersmissen HP Mizoguchi A Vanden Broeck J Orchard I. The distribution and physiological effects of the myoinhibiting peptides in the kissing bug, rhodnius prolixus. Front Neurosci. (2012) 6:98. 10.3389/fnins.2012.00098 22783161 PMC3390896

[78] Hussain A Üçpunar HK Zhang M Loschek LF Grunwald Kadow IC. Neuropeptides modulate female chemosensory processing upon mating in drosophila. PLoS Biol. (2016) 14:e1002455. 10.1371/journal.pbio.1002455 27145127 PMC4856363

[79] Hadfield M Paul V. Natural chemical cues for settlement and metamorphosis of marine-invertebrate larvae. Marine Sci. (2001) 431–61. 10.1201/9781420036602.ch1321558223

[80] Shao L Chung P Wong A Siwanowicz I Kent CF Long X. A neural circuit encoding the experience of copulation in female drosophila. Neuron. (2019) 102:1025–36. 10.1016/j.neuron.2019.04.009 31072787

[81] Kolodziejczyk A Nässel DR. A novel wide-field neuron with branches in the lamina of the drosophila visual system expresses myoinhibitory peptide and may be associated with the clock. Cell Tissue Res. (2011) 343:357–69. 10.1007/s00441-010-1100-7 21174124

[82] Oh Y Yoon S-E Zhang Q Chae H-S Daubnerová I Shafer OT. A homeostatic sleep-stabilizing pathway in Drosophila composed of the sex peptide receptor and its ligand, the myoinhibitory peptide. PLoS Biol. (2014) 12:e1001974. 10.1371/journal.pbio.1001974 25333796 PMC4204809

[83] Schulze J Neupert S Schmidt L Predel R Lamkemeyer T Homberg U. Myoinhibitory peptides in the brain of the cockroach leucophaea maderae and colocalization with pigment-dispersing factor in circadian pacemaker cells. J Comp Neurol. (2012) 520:1078–97. 10.1002/cne.22785 22095637

[84] Stengl M Arendt A. Peptidergic circadian clock circuits in the madeira cockroach. Curr Opin Neurobiol. (2016) 41:44–52. 10.1016/j.conb.2016.07.010 27575405

[85] Takeda N Nakajima Y Koizumi O Fujisawa T Takahashi T Matsumoto M. Neuropeptides trigger oocyte maturation and subsequent spawning in the hydrozoan jellyfish Cytaeis uchidae. Mol Reprod Dev. (2013) 80:223–32. 10.1002/mrd.22154 23341254

[86] Takeda N Kon Y Quiroga Artigas G Lapébie P Barreau C Koizumi O. Identification of jellyfish neuropeptides that act directly as oocyte maturation-inducing hormones. Development. (2018) 145:140160. 10.1101/140160 29358214

[87] Plickert G Schneider B Neuropeptides and photic behavior in cnidaria. Hydrobiologia. (2004) 530–531:49–57. 10.1007/s10750-004-2689-x

[88] Williams EA Verasztó C Jasek S Conzelmann M Shahidi R Bauknecht P. Synaptic and peptidergic connectome of a neurosecretory center in the annelid brain. Elife. (2017) 6:e26349. 10.7554/eLife.26349 29199953 PMC5747525

