# Supplementary material for: Function and Distribution of the Wamide Neuropeptide Superfamily in Metazoans
Source: Front Endocrinol (Lausanne). 2020 May 28;11:344. doi: 10.3389/fendo.2020.00344 (PMC7270403; doi:10.3389/fendo.2020.00344)
Supplement: Supplementary file 1 [file Data_Sheet_1.PDF]

Supplementary Data

List of sequences used to generate alignment of mature Wamide consensus sequences in Figure 2.

Annelida
>XP_009012861.1 hypothetical protein HELRODRAFT_168750 [Helobdella robusta]
MIGQNITITLTFIIISLLHLSINNSLASAASTKNVDNDDNQQYSKLYNDVEENGDNDVTFE
SDKRKWSSNNMGMWGKKRSELDLTPKRAWSSNKMAMWGRKKKSPKIENNGGNDDVI
NDGSSNDNYHNLDEKDLGENAEKREWASNYMGMWGKKRSQMMPPDNLHFPFYIISGN
KRKWIDAGSWMNQPAIQQRRSKWGSGKMMMWGKRSKQGTFNSDDDVIPAPDDV
Eukaryota; Metazoa; Lophotrochozoa; Annelida; Clitellata;Hirudinea; Rhynchobdellida;
Glossiphoniidae; Helobdella.

>ELU01469.1 hypothetical protein CAPTEDRAFT_197880 [Capitella teleta]
MASCRLLLTVITLVICSLVVLADDPETEEQAQDLVPHDMDQDLMDKRKWGSNSMRVW
GKRDGDDEMEMDGGAEKRKWGGNNNMRVWGKRKWGANSMRVWGKRSELPEEEKR
KWGGSNTMRTWGKRADDNEEDELAKRKWGSNSMRVWGKRADDNKRKWGSNSMRV
WGKRADDDMDESKRGWKNNNMRVWGKRADDEIDEDKRKWGSNSMRVWGKRSADD
DAELAAAVPHAIVKRSLDSEEFTDDMEKRRWGGNDMRVWGKRRSRADGPKRSWKTN
VMRVWGKRGWADNNMRVWGKRADEGAEKRAWVGDKSLSWGKRSDNEVIRNLLAE
QVMMISIISPTKYLRDVAICVGGVLGGFSRDPLSE
Eukaryota; Metazoa; Lophotrochozoa; Annelida; Polychaeta; Scolecida; Capitellida;
Capitellidae; Capitella.

>AFV92893.1 MIP/allatostatin B neuropeptide precursor [Platynereis dumerilii]
MDRVTITCFSLCLASVLIPLVHSEENVDLDEDKRAWMKNNIAWGKRGWKQGASYSWG
KRDSEGDGLMSDEEKRAWNKNNMRVWGKRSDEDDKRGWKDSSMRVWGKRAGEDD
NNKRWGKNNLRVWGKRADDLEVLEDKRAWGDNNMRVWGKRSDLEDDKRAWNKNS
MRVWGKRDMEEDEDNKRAWKGQSARVWGKRADEDDKRGWNGNSMRVWGKRGWH
GNGVRQWGKRLHLDDVEPILDDEESKRAWAKNNMRVWGKRSTDNVRNMKAVVAEP
AEVAADAESAKSS
Eukaryota; Metazoa; Lophotrochozoa; Annelida; Polychaeta; Palpata; Aciculata; Phyllodocida;
Nereididae; Platynereis.

Brachiopoda
>XP_013388469.1 protein RNA-directed DNA methylation 3 [Lingula anatina]
MAAITADSLTITFFLTLMVLSSPLVHCVINSQMLRDAQNLDQAPYGRYIEEDIVIPNDSQE
EENSHRYQSQAVSLKRLEYLFNKLENNIMRNEPLKRQWSKSMTMWGKRSPYFYSEDM
EDPHALRLRRRSLEKTDDNNKTKEKTDFQLTNNVIRSWKSGGMKLWGKRPFDKEMEES
RDADLKKLGENMDWKQKERDLKRSWSGNAMKLWGKRDNTFDDQHDKKSWSSKNM
KLWGKREDDKRSWNSKSLKVWGKRDVDQADEENNKRSWSSKNMKLWGKRNEQKDG
LEYEKRPWSSKSMKLWGKRDNSVEADINEKRQWNSNGMKVWGKRMDGEIIDELSNKR
KWNSKEMKLWGKRDSIQHNALNEKRGWNAKGMKLWGKRSEKRPWNGKGMKLWGK
RDSSGYESESNNYPYLYRKRSIDVDSKRSWKSGGIKLWGKRDMADGDVDLVSNLRPAG

GVKRPWNSGMKLWGKKRASDSLRDFDSAKSDSWIQDGKRSWIGKNPWATKIGFDPSK
RWRSMKLWGKRDSGQSQHDYEDALNKWNSMKLWGKDLTSLIKAEIGNNSNTKSLSNE
FEEKVFDHGVDNETNTETGSSPLNKEISNEHVTTRTHTLSKRSGWMPREGLRNMWG
Eukaryota; Metazoa; Lophotrochozoa; Brachiopoda; Linguliformea; Lingulata; Lingulida; Linguloidea; Lingulidae; Lingula.

Mollusca
>NP_001191611.1 PTSP-like peptide neurotransmitter precursor [Aplysia californica]
MTLHLASPFILLFTIAYSLTSAVQGLEPLPAASLSDSPASGADVPPLPSSAATNAAVDKE
WLRQKLEEGQFLPQQDKRWGGINSWMTHRLGGPSERDSSQDSLDKQLLVNNVQNYDD
SSKRKWSKFSSWGKRDASEETPEGGEGEDGLGAVKKWKNMAVWGKRAEDGLDKRW
KQMATWGKREDGDVLGLGTDKRWKQMASWGKRLDDSDRDKKWKQMSVWGKRED
NGEPLDKKWKEMSVWGKRDTLDDPEKRWKQMAVWGKRQGLDDRNDKRWKQMAT
WGKRNSSENYDKRWKQMSVWGKRDGDGDLDKRWKQMSVWGKRDGDGDLDKRWK
QMSVWGKRNGDGDLDKRWKQMSVWGKRDGDEDVEKRWKQMSVWGKRDGDADLD
KRWKQMSVWGKRDEDGNLDKRWKQMSVWGKRDEDGNLDKRWKQMSVWGKRDGD
DNLDKRWKQMSVWEREREREWV
Eukaryota; Metazoa; Lophotrochozoa; Mollusca; Gastropoda;Heterobranchia; Euthyneura; Euopisthobranchia; Aplysiida;Aplysioidea; Aplysiidae; Aplysia.

>XP_009051899.1 hypothetical protein LOTGIDRAFT_231501 [Lottia gigantea]
MDLKTILCLIIYSLLLQISHAEEQLANDIELSNSLNPVDKRAWKSSYLNTWGKRWNPRYN
LRGYQRMPIWAKRWTNSGLITWGKRSADTEIPIHKRKWNQFITWGKRSGVPSIVKRSVG
DELVPWGKNKDTLPELNTSSDNLDNKLIDLETTPSTDKLSLDEKRASDKGWNGFTTWG
KRANKDWSSLSTWGKRGQNKDWSSLTTWGKRGHDRDWNSLTTWGKRANKDWSSLS
TWGKRARENDWSALSTWGKRANNKDWASLTTWGKRANDRDWNSLTTWGKRAKGN
NWSGLTTWGKRANKDWNSLTTWGKRANKDWSGLTTWGKRGNKDWSGLTTWGKRG
NKDWSGLTTWGKRGNKDWSGLTTWGKRGNKDWSGLTTWGKRGKNDWSGLTTWGK
RGNKDWSGLTTWGKRGNNDWSGLTTWGKRGKNDWSGLTTWGKRSPDATSEDSGELS
TLDKKDIKGWNGLTTWGKRFAGDKNKWSSLTTWGKRDDNNKQDDKKWAQLSTWGK
RSPEDAAELWKIYDSNGDGIMDKEEMVSFLRSAASQKDSQQDEKS
Eukaryota; Metazoa; Lophotrochozoa; Mollusca; Gastropoda; Patellogastropoda; Lottioidea; Lottiidae; Lottia.

>XP_011417556.1 PREDICTED: uncharacterized protein LOC105321065 [Crassostrea gigas]
MLCHLEQLLLSCIVLCLVKVCASLKAQDEASVNDHDIVRRQAMGHSFGDELEGFDLDP
KRVNNWNQFPAWGKRLSKRRWSSLGAWGKRSWLDRLISANNNWGKRWKSMSNSWG
KRQAPSEFDGLSDDYINIKKRSVDSKFSHNSRNKRSIPTELSPEQNEEKRRWSSLSAWGK
RSDDDEKRRWSSLSAWGKRSNPEAIDDNDSDNISKRKWSSFSSWGKRGDPVDLSKRLY
SYWQNRLMTNNPWMERRGWNAFSSWGKRSMD
Eukaryota; Metazoa; Lophotrochozoa; Mollusca; Bivalvia;Pteriomorphia; Ostreoida; Ostreoidea; Ostreidae; Crassostrea.

>XP_014775853.1 PREDICTED: uncharacterized protein LOC106873140 [Octopus bimaculoides]

MATFLNFLLFVTVLALTKFSTLSAELNELSNKQSAIKVARSMMPAKFLESDLEAWDEKI
GDQRNWEDRLNMKRNWDELSSWGKRSAFDNNAIDNEFGNSLRRALNSHNLKLSSLLN
GAELQKRWDSLQAWGKRNANGNEAAYGKNVKRDHMVKASDGTWKKVGSADGDKR
GNGWDEMNGWGKRNAVQHKIRSRNWDSLQAWGKRESPSDDSALYENNQRQKRSTGK
IL
Eukaryota; Metazoa; Lophotrochozoa; Mollusca; Cephalopoda;Coleoidea; Neocoleoidea; Octopodiformes; Octopoda; Incirrata;Octopodidae; Octopus.

Arthropoda
>KZS20495.1 AST-B/myoinhibitory peptide-like protein [Daphnia magna]
MQLWQCPLLLMVSLIAAINTQQTPSQQRFEPNQVAELVELHQQLGPSREQQEQPAQAD
NKDHQLAASPAALLQLTPSDWRSSEDKRNWNRMQGMWGKRSQQQSDESATLHDMTP
QRMVKRAWSDLSQQGWGKRSWTQLHGVWGKRRWDQLHGAWGKRTVDGQVDSQED
QSAETEREDVSDSDELETSKRSGWNKMQGVWGKRSDTPNLDGIGNNDLLLMLSGDQL
YLKDDDQPTAKLVEDDENGESTNKRAWNQMQGVWGKRALSAMAAGYKRNWNNLRG
AWGKREIPAAVAKGMEWSRKRESGWNNLKGLWG
Eukaryota; Metazoa; Ecdysozoa; Arthropoda; Crustacea; Branchiopoda;Diplostraca; Cladocera; Anomopoda; Daphniidae; Daphnia.

>XP_018024231.1 PREDICTED: prothoracicostatic peptide-like [Hyalella azteca]
MGWVRIRENISIVRFMVILSLVWTSLASEQSIHKSTNNFGPANQSFLSRVTSEKSAVADG
GELNVYGTSSEDHVAQEAEGEPNLLIDDELRTKRDGEKWSSLRGSWGKRNPAQLDRG
WNNFRGTWGKKADWSSLRGSWGKRGSDAMADKRADWSNFRGSWGKRTSAPEASLD
DSVSDLDQFDHDYDAFNEDPSSALSPDDATRLNNDALFLETVKLLSQRAQNKEDFADQ
EILNSGDDEEQNLVQKRSTAAYSGWLKRPVGTLLLAPRSVNWSSLRGSWGKRSGPTNW
SSLRGTWGKRPAKDSNWAGLRGTWGKRPAKDSNWADLRGTWGKRPAKDSNWANLR
GTWGKRPAKDSNWANLRGTWGKRPAKDSNWADLRGTWGKKSGGQKWSSLRGTWG
KRSDQVDELDLGDE
Eukaryota; Metazoa; Ecdysozoa; Arthropoda; Crustacea; Malacostraca; Eumalacostraca; Peracarida; Amphipoda; Senticaudata; Talitrida;Talitroidea; Hyalellidae; Hyalella.

>ATQ64324.1 B-type allatostatin [Megabalanus volcano]
MWTRCLLLSWACLLLLLTVRAGSAEAEQPQAVTAGKRDWDALHGNWGKRQPSEVIDD
PEDDRELMREVAGDLVLSGYEPEDKRKWNSFTGSWGKRAKWNNFSGSWGKRSKWNS
MGPSWGKRAKWDNFGGSWGKRGKWENFGGSWGKRKNWDNFNGAWGKRKGWDSFS
GGWGKRAAWNNFGGSWGKRDASLPTLQEASDPLETFIKNNYKLLQARVPASSGQTQA
RKRKDWTQLNGMWGKRSPAKELLLV
Eukaryota; Metazoa; Ecdysozoa; Arthropoda; Crustacea; Maxillopoda;Cirripedia; Thoracica; Sessilia; Balanidae; Megabalanus.

>NP_001137202.1 allatostatin type b precursor [Tribolium castaneum]
MMSFAAAIMRDAVAPVLGAVLLTCYSLQATLALSDETPLKSSNDNPQIEDEMSKRDWN
KDLHIWGKRGWNNLHEGWGRKRSVPAWEEQQEKRAWQSLQSGWGKRFAPEDEYAIR
QLAAMLDSQYDDYNPEIETNDDEKRNWGQFHGGWGKRSKWDNFRGSWGKREPAWSN
LKGIWGKRSGEK

Eukaryota; Metazoa; Ecdysozoa; Arthropoda; Hexapoda; Insecta; Pterygota; Neoptera; Holometabola; Coleoptera; Polyphaga; Cucujiformia; Tenebrionidae; Tenebrionidae incertae sedis; Tribolium.

>NP_001036890.1 prothoracicostatic peptide precursor [Bombyx mori]
MRWCLFALWVFGVATVVTAAEEPHHDAAPQTDNEVDLTEDDKRAWSSLHSGWAKRA
WQDMSSAWGKRAWQDLNSAWGKRGWQDLNSAWGKRAWQDLNSAWGKRGWQDLN
SAWGKRDDDEAMEKKSWQDLNSVWGKRAWQDLNSAWGKRAWQDLNSAWGKRGW
NDISSVWGKRAWQDLNSAWGKRAWQDMSSAWGKRAPEKWAAFHGSWGKRSSIEPD
YEEIDAVEQLVPYQQAPNEEHIDAPEKKAWSALHGTWGKRPVKPMFNNEHSATTNEA
Eukaryota; Metazoa; Ecdysozoa; Arthropoda; Hexapoda; Insecta;Pterygota; Neoptera; Holometabola; Lepidoptera; Glossata; Ditrysia;Bombycoidea; Bombycidae; Bombycinae; Bombyx.

>XP_021915053.1 prothoracicostatic peptide [Zootermopsis nevadensis]
MEYFAIPGVILWLLLLAVSPSSQGDPISDPARVPPGSGPSGGTLSRAQDVPTQVQGPEED
KRGWRDLQGGWGKRGWQDLQIPYSHSPEEMEYFAIPGVILWLLLLAVSPSSQGDPISDP
ARVPPGSGPSGGTLSRAQDVPTQVQGPEEDKRGWRDLQGGWGKRGWQDLQGGWGKR
GWQDLQGGWGKRGWQDLQGGWGKRGWQDLQGGWGKRGWQDLQGGWGKRGWQD
LQGGWGKRGWQDLQGGWGKRGWQDLQGGWGKRGWQDLQGGWGKRGWQDLQGG
WGKRGWQDLQGGWGKRGWDKFHGSWGKRDSDLDFDVNSLDNDVADEELVDEESGE
DLKRAWSSLKGGWGKRAADWANFRGSWGKRDPGWNNLKGLWGKRADTNWNRLSA
AWGKRSIGGETGIKEDPARVMSSSEE
Eukaryota; Metazoa; Ecdysozoa; Arthropoda; Hexapoda; Insecta;Pterygota; Neoptera; Polyneoptera; Dictyoptera; Blattodea;Blattoidea; Termitoidae; Termopsidae; Zootermopsis.

>XP_014260688.1 prothoracicostatic peptide isoform X2 [Cimex lectularius]
MSWRTLLFLAWVAVAINCEVPDFVGPQSPLDEETNSALNLPEDSPNKKAWKDLTTAWG
KRGWKDLPATGWGKRGWQDLQSAWGKRGWTDLNSGGWGKRGWQDLNSGGWGKR
GWQDLNSGGWGKRGWQDLQAPAWGKREEKSLDEKESEGEDAKRSWVSLHSGWGKR
AADWGSFRGSWGKKDPAWQNLKGLWGKRNYNGFMEPYDPGYNTLDEEGRRAK
Eukaryota; Metazoa; Ecdysozoa; Arthropoda; Hexapoda; Insecta;Pterygota; Neoptera; Paraneoptera; Hemiptera; Heteroptera;Panheteroptera; Cimicomorpha; Cimicidae; Cimex.

>AAF49354.1 myoinhibiting peptide precursor [Drosophila melanogaster]
MAHTKTRRTYGFLMVLLILGSACGNLVASGSAGSPPSNEPGGGGLSEQVVLDQLSESDL
YGNNKRAWQSLQSSWGKRSSSGDVSDPDIYMTGHFVPLVITDGTNTIDWDTFERLASG
QSAQQQQQQPLQQQSQSGEDFDDLAGEPDVEKRAWKSMNVAWGKRRQAQGWNKFR
GAWGKREPTWNNLKGMWGKRDQWQKLHGGWGKRSQLPSN
Eukaryota; Metazoa; Ecdysozoa; Arthropoda; Hexapoda; Insecta; Pterygota; Neoptera; Holometabola; Diptera; Brachycera; Muscomorpha; Ephydroidea; Drosophilidae; Drosophila; Sophophora.

>XP_021196035.1 prothoracicostatic peptide-like isoform X1 [Helicoverpa armigera]

MRYCVAALWLSALITLVVAAADDAHHDVAPAQNDNEIELTEEEKRAWSSLHGGGGWG
KRGWQDMSSAWGKRGWNDMSSAWGKRGWNDMSSAWGKRAWQDLNSAWGKRGW
QDLNSAWGKRAWRDMSQSPWGKRGWQDMSSAWGKRGWNDMSSAWGKRGWNDMS
SAWGKRGWNDMSSAWGKRGPEKWANFHGSWGKRSEDPDYEEIDAAIEQLIPIQQLSES
ERMDTPEKKAWSALHGAWGKRPVKQAQYNSGSYYWKREPAWTNLRGMWGKRSGQV
DADAVDDDHESSARDEA
Eukaryota; Metazoa; Ecdysozoa; Arthropoda; Hexapoda; Insecta; Pterygota; Neoptera; Holometabola; Lepidoptera; Glossata; Ditrysia; Noctuoidea; Noctuidae; Heliothinae; Helicoverpa.

>AKN21242.1 allatostatin-B precursor, partial [Locusta migratoria]
AWQDLGSAWGKRAWQDLGSAWGKRAWQDLNAGWGKRAWQDLGSAWGKRAWQDL
NAGWGKRGWRDLQSAWGKRAWSNLNGAWGKRGGDDEATWPELPDQTITEDEDGDH
DLMQMQMPLPLAMQLQGDEGEPGDEQKRAWSSLHGAWGKRAADWRAFHGSWGKRE
PGWTNLKGLWGKRAGPSNWNRLPAVWGKRSEDE
Eukaryota; Metazoa; Ecdysozoa; Arthropoda; Hexapoda; Insecta; Pterygota; Neoptera; Polyneoptera; Orthoptera; Caelifera; Acrididea; Acridomorpha; Acridoidea; Acrididae; Oedipodinae; Locusta.

>XP_013784813.1 prothoracicostatic peptide-like [Limulus polyphemus]
MKFPTYGFCILFIFHVVIQIQSKPTDTDMTLDDALANVENEAGDKRGWNNLSGMWGKR
GWNNLSGMWGKRGSSWNDLSGMWGKRGWNNLSEILGKRSSGWNNLSGMWGKRGW
NNLSGMWGKRSSKWNNLKGLWGKRAISPLDEERIAWDIVESDNFPSVNHQLLANILSE
D
Eukaryota; Metazoa; Ecdysozoa; Arthropoda; Chelicerata; Merostomata; Xiphosura; Limulidae; Limulus.

>XP_030023380.1 prothoracicostatic peptide-like [Manduca sexta]
MRCCVAVLWAFFATWAVAAAEEPHHDAAPQTDNELDLTDEDKRAWTSLRGGWAKRG
WQDMSSAWGKRAWQDLNSAWGKRAWQDLNSAWGKRAWQDLNSAWGKRGWQDLN
SAWGKRSDDEAMDKRAWQDLNSAWGKRGWQDMSSAWGKRAWQDLNSAWGKRGW
NDMSSAWGKRAWQDLNSAWGKRGWQDMSSAWGKRAPEKWAAFHGSWGKRAAEPD
YEELDAAIEQLVPIHQMDEDRMDAPEKKAWSALHGAWGKRPVKQAQFNSGAYYWKR
EPGWTNLRGMWGKRSAPFEEALTDDHESSAARDEA
Eukaryota; Metazoa; Arthropoda; Hexapoda; Insecta; Pterygota;Neoptera; Endopterygota; Lepidoptera; Glossata; Ditrysia;Bombycoidea; Sphingidae; Sphinginae; Sphingini; Manduca.

Tardigrada
>OWA51718.1 hypothetical protein BV898_16190 [Hypsibius dujardini]
MNYLMAFVCIALVAFLGSVLCVPAAVAPAKDSVALKAADEPSEHNDIIVKRDWNGMR
AAWGKRADDYADLEEELPERQRRGPSSSWQSGMGAWGKRANDWGSMRAAWGKRTP
TEWNPEYIQTLQRQMEAVKNRRAGTWQLASWGRRR
Eukaryota; Metazoa; Ecdysozoa; Tardigrada; Eutardigrada; Parachela; Hypsibiidae; Hypsibius.

>GAU98064.1 hypothetical protein RvY_09262 [Ramazzottius varieornatus]

MEKIVSLSGLCLLGFVLLGSFAPKTSAQSTAAISTGMDKAEKRDWADMKSSWGKRSQE
PDDITVEDLQLAEIPTRSIREAAGWRDGMRASWGKRADRDVSNGWTSGLKAWGKRGA
WSQNLRAWGKRTPVEWTDEYVRSLQRQIAAAKNRRASNWQLASWGK
Eukaryota; Metazoa; Ecdysozoa; Tardigrada; Eutardigrada; Parachela; Hypsibiidae; Ramazzottius.

Nematoda
>NP_001252155.1 Neuropeptide-Like Protein [Caenorhabditis elegans]
MQLIHFIVGLAMLISLSLAASDDRVLGWNKAHGLWGKRSVQEASQDKRTPQNWNKLN
SLWGKRSASSFDDDYTTENGDDDVTMLYKRSPAQWQRANGLWGR
Eukaryota; Metazoa; Ecdysozoa; Nematoda; Chromadorea; Rhabditida; Rhabditina; Rhabditomorpha; Rhabditoidea; Rhabditidae; Peloderinae; Caenorhabditis.

>KHN83875.1 hypothetical protein Tcan_11366 [Toxocara canis]
MMCSQSSLALQMLRAGIVLLVVIVSLSPPSTLAYEIDKRNNWNKAVGLWGKRSIPSQLL
DSGLEKRPQNQWNKLNSLWGKRSSWQTANGLWGKRSSWQTANGLWGKRSLRFEELD
GYH
Eukaryota; Metazoa; Ecdysozoa; Nematoda; Chromadorea; Ascaridida; Ascaridoidea; Toxocaridae; Toxocara.

Priapulida
>XP_014663362.1 PREDICTED: prothoracicostatic peptide-like [Priapulus caudatus]
MQMTCCHCYLLLLAGLAALALALAEDTNELGNAVDIDAPHDDKRGWHDLGHAYGKR
AGWHDLQTSWGKRDNDADEQEKRGWHDLDTAFGKRGWRDLNTAFGKRGWRDLNTA
FGKRGWRDLRMAFGKRDAVDTAEDQLVADEEKRGWHDLNTAFGKRGWRSLNSAFGK
RGDNSAAIIEDLITEQLFDDIDSDGDKCIDRKELRLLVKKLLAGEL
Eukaryota; Metazoa; Ecdysozoa; Scalidophora; Priapulida; Priapulidae; Priapulus.

Cnidaria
>AYD75757.1 GLWamide precursor [Nematostella vectensis]
MALFGHTLVAVLFCLALCHAETKRKAADTTDTENELDASPNVNNDDDNDIKRMPTDTK
RQAGAPGLWGKRDAGPPGLWGKRDAGPPGLWGKRSPKPPGLWGKRQAGAPGLWGK
RSAGPPGLWGKRDAGPPGLWGKRVAGPPGLWGKRQAGAPGLWGREAGAPGLWGKR
QAGAPGLWGKREAGAPGLWGREANAPGLWGKRRAGAPGLWGKREANAPGLWGKRQ
AGPPGLWGKREANAPGLWGKRQAGPPGLWGKREANAPGLWGKRQAGPPGLWGKRD
EDEDEDMDETNGDPLWGRSADAGPPGLWGRKKRAASPQRDLYGIGLWGRNAALMTA
EELDLSFKNEEQS
Eukaryota; Metazoa; Cnidaria; Anthozoa; Hexacorallia; Actiniaria;Edwardsiidae; Nematostella.

>XP_002164748.1 PREDICTED: LWamide neuropeptides [Hydra vulgaris]
MGMFERKKIVLLVSLICVSQQATNVQDANSKSTSTELKVVKPQKRVTPVKDAEKLSILR
TQDNSLDLNTNGEEVWDELTHNIPLEYIEKIYNELNQLAQNENRPKRLWGATAAINTDN
LNPEVENELENKKNAPVIEKFERPIGLWHKDIETKNPENRLPLGLWGKDSEPLPIGLWGK
DADVNDDLKKEPLPIGLWGKDTDSTRGDNKPNAYKGKLPIGLWGKDNALTNDLGKKN
NGKDSGPPPGLWGKDSKPIPGLWGKDNGPMTGLWGKKDVGPPPGLWGKKDQPPIGM

WGRAGKRDSNPYPGLWGKKEEELENVDKEIEEDSLEEFPACLLENPPCEIQEKRYNIDKS
GPPPGLWGKRSEKYQMNKPPWRGGMWGRSEILENSVHDSKKTNTIDMEHAEN
Eukaryota; Metazoa; Cnidaria; Hydrozoa; Hydroidolina; Anthoathecata; Aplanulata; Hydridae; Hydra.

>ALY05321.1 peptide precursor 2 [Clytia hemisphaerica]
MKIYFGCLFVILSVNQIGCYPSSNQNSERELVRRIYKTVHPNPHYQVNEIQRVKEALKRR
VLENVHRVDSLKASLKRVLGQDAGNGFHLSSSKIFQKKRSKARLPHSYMFRKRQNSPG
ALGLWGREVEAPGDIGPPGIWGDVVPDETRKDKPGAVQGLWGKDERVIRALLKTLKR
Eukaryota; Metazoa; Cnidaria; Hydrozoa; Hydroidolina; Leptothecata;Campanulariidae; Clytia.

>ARN59429.1 pp11 precursor [Clytia hemisphaerica]
MDQSLSSILLLLCCWVALTTCMSVQRKEAGDALSALDKENAKKSANSITEELARNLME
HLYDEIRKRSNSNEETISNFRRASSDTHRQQQAPKGLWGRELQPGNPPGLWGREASEAE
NTDSNDGPIPGMWGRREADDKNAHEKFQ
Eukaryota; Metazoa; Cnidaria; Hydrozoa; Hydroidolina; Leptothecata;Campanulariidae; Clytia.

>sp|Q16992.1|LWA_ANTEL RecName: Full=LWamide neuropeptides; Contains: RecName: Full=LWamide I; Contains: RecName: Full=Metamorphosin A; Short=MMA; AltName: Full=LWamide II; Contains: RecName: Full=LWamide III; Contains: RecName: Full=LWamide IV; Contains: RecName: Full=LWamide V; Contains: RecName: Full=LWamide VI; Contains: RecName: Full=LWamide VII; Contains: RecName: Full=LWamide VIII; Contains: RecName: Full=LWamide IX; Flags: Precursor
MALKCHLVLLAITLLLAQCSGSVDKKDSTTNHLDEKKTDSTEAHIVQETDALKENSYLG
AEEESKEEDKKRSAAPQQPGLWGKRQKIGLWGRSADAGQPGLWGKRQSPGLWGRSAD
AGQPGLWGKRQNPGLWGRSADAGQPGLWGKRQNPGLWGRSADAGQPGLWGKRQNP
GLWGRSADARQPGLWGKREIYALWGGKRQNPGLWGRSADPGQPGLWGKRELVGLW
GGKRQNPGLWGRSAEAGQPGLWGKRQKIGLWGRSADPLQPGLWGKRQNPGLWGR
SADPQQPGLWGKRQNPGLWGRSADPQQPGLWGKRQNPGLWGRSADPQQPGLWGKRQ
NPGLWGRSADPQQPGLWGKSPGLWGRSADPQQPGLWGKRQNPGFWGRSADPQQPGL
WGKRQNPGLWGRSADPQQPGLWGKRQNPGLWGRSADPQQPGLWGKRQNPGLWGRS
ADPQQPGLWGKRQNPGLWGRSAGSGQLGLWGKRQSRIGLWGRSAEPPQFEDLEDLKK
KSAIPQPKGQ
Eukaryota; Metazoa; Cnidaria; Anthozoa; Hexacorallia; Actiniaria;Actiniidae; Anthopleura.

Placozoa
>RDD46844.1 Antho-RFamide neuropeptides type 2 [Trichoplax sp. H2]
MLTNRFIIWILFLGITTAQNVAKGKAQIGNHKSVFLKNEATRPERDQPPRWGRDQPTRW
GRDQPPRWGRDQPPRWGRDQPSRWGRDQPPRWGRDQPPRWGRDQPPRWGRDQPPRW
GRDQPPRWGRDQPPRWGRDQPPRWGRDQPPRWGRDQPPRWGGDQLPEIEKNYAPPR
WGRDQYSWWNQEQYPSRWGREYSTPDNTAEKLLDSLTHQSENAKKNNFQEINSDSNS
GNESAVHRLFSNKLKNQKAKSDSNKLMNSFSGSESISRPREKSLKRSETLDNMRIDLI
Eukaryota; Metazoa; Placozoa; Trichoplax.
